# Lithium prescription trends in psychiatric inpatient care 2014 to 2021: data from a Bavarian drug surveillance project

**DOI:** 10.1186/s40345-023-00323-6

**Published:** 2023-12-19

**Authors:** Paul Kriner, Emanuel Severus, Julie Korbmacher, Lisa Mußmann, Florian Seemueller

**Affiliations:** 1 Kbo-Lech-Mangfall-Klinik Garmisch-Partenkirchen, Auenstrasse 6, 82467 Garmisch-Partenkirchen, Germany; 2 Asklepios Klinik Nord Psychiatrie Ochsenzoll, Langenhorner Chaussee 560, 22419 Hamburg, Germany; 3 Bayerisches Institut Für Daten, Analysen Und Qualitätssicherung, Am Moosfeld 13, 81829 Munich, Germany; 4 Department of Psychiatry and Psychotherapy Nussbaumstrasse 7, Ludwig-Maximilians-Universität, 80336 Munich, Germany

**Keywords:** Lithium, Prescription rate, Bipolar disorder, Unipolar depression, Schizoaffective disorder, Polypharmacy, Drug–drug interactions, Comorbid substance use disorder, Elderly patients

## Abstract

**Objectives:**

Lithium (Li) remains one of the most valuable treatment options for mood disorders. However, current knowledge about prescription practices in Germany is limited. The objective of this study is to estimate the prevalence of current Li use over time and in selected diagnoses, highlighting clinically relevant aspects such as prescription rates in elderly patients, concomitant medications, important drug–drug interactions, and serious adverse events.

**Methods:**

We conducted a descriptive analysis of Li prescriptions, analyzing data from the ongoing Bavarian multicenter drug safety project Pharmaco-Epidemiology and Vigilance (Pharmako-EpiVig) from the years 2014–2021. Our study included 97,422 inpatients, 4543 of whom were prescribed Li.

**Results:**

The Li prescription rate in unipolar depression (UD) remained constant at 4.6% over the observational period. In bipolar disorder (BD), the prescription rate increased significantly from 28.8% in 2014 to 34.4% in 2019. Furthermore, 30.3% of patients with Li prescriptions did not have a diagnosis of BD or UD, and 15.3% of patients with schizoaffective disorder were prescribed Li. The majority (64%) of patients with Li prescriptions were prescribed five or more drugs. Most of the 178 high-priority drug–drug interactions were due to hydrochlorothiazide (N = 157) followed by olmesartan (N = 16).

**Conclusion:**

Our study does not substantiate concerns about a decline in Li prescription. The decline in prescription rates observed in some diagnostic groups in 2020 and 2021 may be associated with the COVID-19 pandemic. The symptom-oriented use of Li beyond BD and UD is common. Polypharmacy and drug–drug interactions present a challenge in Li therapy. Old age and comorbid substance use disorder do not appear to be major deterrents for clinicians to initiate Li therapy.

**Supplementary Information:**

The online version contains supplementary material available at 10.1186/s40345-023-00323-6.

## Background

Lithium (Li) has been a valuable drug for treating mood disorders since its antimanic effects were first described by Cade over 70 years ago. Since then, research has highlighted additional benefits of Li, including its exceptional efficacy as a mood stabilizer and unique anti-suicidal properties (Bauer and Gitlin 2016a; Bauer et al. 2006; Cipriani et al. 2013; Lewitzka et al. 2015). In bipolar disorder (BD), Li is consistently considered first-choice therapy for long-term mood stabilization (Tondo et al. 2019; DGBS e.V. und DGPPN e.V. 2019; Fountoulakis et al. 2022). Li is also recommended for augmentation in treatment-resistant unipolar depression (UD) and long-term prophylaxis in recurrent UD (Bundesärztekammer (BÄK) et al. 2022; Abou-Saleh et al. 2017). Despite these indications, Li is often used for non-approved psychiatric indications, including schizophrenia (SCZ) and schizoaffective disorder (SAD), for which solid evidence is lacking (DGPPN e.V. (Hrsg.) 2019).

Side effects such as nephrotoxicity, tremor, and weight gain, as well as interactions with comedications, need to be considered when prescribing Li (Gitlin 2016a). Prescription practices for Li vary widely internationally, with notably low prescription rates in BD in the United States (Pérez de Mendiola et al. 2021; Rhee et al. 2020; Singh et al. 2023). In Germany, some studies have shown a decline in Li use in BD (Greil et al. 2012; Bohlken et al. 2020), while analyses of outpatient prescription data in German Statutory Health Insurance show a slight incline in overall Li prescription over the past decade (Schwabe and Arzneiverordnungsreport 2010, 2020).

The Pharmacoepidemiology and Pharmacovigilance (Pharmako-EpiVig) Project, conducted by the Bavarian Institute for Data, Analysis and Quality Assurance (BIDAQ), has been gathering inpatient prescription data from 26 Bavarian hospitals since 2014 (Pharmako-Epidemiologie und -Vigilanz 2023). By assessing Li prescription patterns in various diagnosis groups, we hope to provide valuable insights into the current use of Li in clinical practice and identify areas for improvement.

The objective of this study was therefore to assess Li prescription patterns in diagnosis groups with guideline recommendations for Li utilization, as well as in groups for which there is no guideline recommendation or evidence for efficacy of Li. We also evaluated Li prescription rates in vulnerable groups such as young women, older patients, and patients with comorbid substance abuse. Additionally, we assessed the prevalence of somatic comorbidities that constitute (relative) contraindications to Li use and the prevalence of polypharmacy, with an emphasis on comedications with a risk of drug–drug interactions with Li. Lastly, we characterized all severe adverse drug reactions (sADRs) attributed to Li, reported in the Pharmako-EpiVig surveys.

## Methods

### Data source

Since 2014, the ongoing Pharmako-EpiVig project collects data on two reference days a year in a cross-sectional approach. Up to 26 psychiatric hospitals in Bavaria are participating in the survey. A list with all participating hospitals can be found in Additional file 1. All inpatients being treated at the participating hospitals on the reference days are included in the survey (Pharmako-Epidemiologie und -Vigilanz 2023). Patients’ year of birth, sex, ICD-10-codes of main conditions treated during the hospital stay, i.e. principal psychiatric diagnoses, and of secondary psychiatric as well as somatic diagnoses, along with all drugs administered that day, including daily dosages are reported. In addition, sADRs that occurred within two weeks before the reference day are reported. The questionnaire can be found in Additional file 1. To improve data quality, BIDAQ performs return control and data cleansing procedures. Furthermore, BIDAQ also evaluates drug–drug interactions of prescribed medication using the internet-based drug–drug interaction program mediQ (mediQ-Interaktionsdatenbank 2023). The dataset analyzed for this study includes inpatients from departments of general psychiatry, addiction medicine, geriatric psychiatry, psychosomatic medicine and affiliated departments in participating Bavarian district hospitals from 2014 to 2021.

The study protocol and analysis has been approved by the ethics review committee of the Technical University Munich (TUM).

### Study population and design

Between 2014 and 2021 a total of 97,422 patients were included in the survey.

We described the study population by age, sex and the ten most prevalent principal psychiatric diagnoses according to the International Classification of Disease in its 10th Version, German Modification (ICD-10-GM) (Internationale statistische Klassifikation der Krankheiten und verwandter Gesundheitsprobleme 10,2023). Diagnoses were categorized using the three-character code of the ICD-10 classification system.

We evaluated total prescription numbers and prescription rate of Li in patients with principal diagnosis of BD (defined as ICD-10-code F31, excluding F30), UD (ICD-10-codes F32 or F33), SAD (ICD-10-code F25) and SCZ (ICD-10-code F20) by year and in total. We also sought to investigate whether sex and age-related issues such as childbearing age or age-related toxicity might influence prescription trends. Therefore, we compared prescription rates by sex in patients younger than 41. Additionally, we compared prescription rates of patients younger than 65 with prescription rates of patients older than 65. Moreover, we checked for differences in patients with comorbid substance use disorder, defined as additional diagnosis of mental and behavioral disorders due to psychoactive substance use, excluding tobacco use (ICD-10-codes F10–F19, excluding F17).

Comorbid somatic diagnoses in the Li group were analyzed for relative or absolute contraindications for Li prescription. In accordance with recommendation for Li use in the 2019 German S3 guidelines for BD, absolute contraindications were defined as acute renal failure (ICD-10-code N17) and acute or subsequent myocardial infarction (ICD-10-codes I21 and I22). Relative contraindication was defined as chronic kidney disease (ICD-10-code N18), unspecified kidney failure (ICD-10-code N19), psoriasis (ICD-10-code L40), primary adrenocortical insufficiency (ICD-10-code E27.21) and Addisonian crisis (ICD-10-code E27.2) (DGBS e.V. und DGPPN e.V. 2019).

Mean numbers of drugs prescribed per person were analyzed in the Li and non-Li group. We also assessed polypharmacy in both groups, with polypharmacy being defined as simultaneous prescription of five drugs or more (Masnoon et al. 2017). A mediQ-check was performed for Li comedications to identify drugs with intermediate-priority and high-priority drug–drug interactions (mediQ-Interaktionsdatenbank 2023).

All prescribed drugs with high-priority drug–drug interactions with Li, were presented along with description of interaction potential.

Finally, all sADRs with Li as the reported probable causative agent, that occurred from 2016 to 2021 were identified and described, along with additional information about comedication and measures taken because of the adverse reaction. The definition of sADRs comprised the GCP-criteria and sADRs that were defined in concordance with the Working Group for Pharmaceutical Treatment of Psychiatric Diseases (Arbeitsgemeinschaft für Arzneimitteltherapie bei psychiatrischen Erkrankungen, AGATE, Table 1) (Definition einer schweren unerwünschten Arzneimittelwirkung (sUAW) bei AMÜP, AGATE 2023). For the analysis of sADRs data from 2014 and 2015 were excluded because the reporting of probable causative agents was not included in the sADR reporting system during those years. We also excluded four reported sADRs, in which no measures were taken because of the adverse reaction, due to incompatibility with above stated definitions.

**Table 1:** Definition of serious and specific serious adverse events

Definition of serious adverse events	Definition of specific serious adverse events
• Event leads to hospital admission • Event leads to extension of the inpatient stay • Event leads to death • Event results in permanent damage • Event prompts the treating physicians to discontinue a medication	• Hypertension with blood pressure values > 200 mmHg systolic or > 120 mmHg diastolic • Collapse, if accompanied by actual sudden falling • Cardiac arrhythmias or conduction disorders: - If they lead to transfer to an internal medicine department - If they are considered severe from an internal medicine perspective - If QT interval prolongation > 470 ms in women, > 450 ms in men - If there is an increase of > 60 ms after the start of treatment - Tachycardia with a heartrate of > 120 beats per minute or clinical symptoms • Agranulocytosis, with granulocytes < 500 cells/μl • Neutropenia, with neutrophils < 1500 cells/μl • Leukopenia, with leukocytes < 3000 cells/μl

Definition of serious adverse events according to GCP-criteria and specific serious adverse events that were defined in concordance with the Working Group for Pharmaceutical Treatment of Psychiatric Diseases (Arbeitsgemeinschaft für Arzneimitteltherapie bei psychiatrischen Erkrankungen)

### Statistical analysis

Due to the naturalistic data and non-hypothesis-based nature of this study, the analysis was descriptive. Data are presented as percentages for categorical variables and as means and standard deviations for continuous variables. Apart from usual descriptive statistics, Chi-square tests and t-tests were applied as appropriate to screen for differences between Li and Non-Li Patients. Chi-square test was used to test for significant differences in Li prescription numbers in selected years. Differences were reported as statistically significant if the p-value was less than or equal to 0.05. Time trends in development of relative frequencies in Li prescription in relevant diagnosis groups over the observation period were demonstrated in a line chart.

Relative risk (RR) along with 95% confidence interval (95% CI) was used to compare differences in prescription numbers in selected groups. RRs were reported as statistically significant, when null value of one was not included in the 95% CI.

## Results

### Characteristics of study population and patients with lithium prescription

4543 patients, representing 4.7% of the study population were administered Li. Mean Li dosage administered was 21.28 mmol with a standard deviation of 8.41 mmol. Characteristics of the study population and patients with Li prescription are shown in Table 2.

**Table 2:** Characteristics of study population and patients with and without lithium prescription

N	Study population	Patients with Li prescription	Patients without Li prescription
	97,422	4543	92,879
Age in years			
≤ 30	19,202 (19.7%)	728 (16.0%)	18,474 (19.9%)
31–60	52,492 (53.9%)	2782 (61.2%)	49,710 (53.5%)
> 60	25,728 (26.4%)	1033 (22.7%)	24,695 (26.6%)
Mean	49.2 (SD 18.9)	48.7 (SD 15.6)	49.2 (SD 19.1)
Sex			
Male	47,223 (48.5%)	1975 (43.5%)	45,248 (48.7%)
Female	50,114 (51.4%)	2563 (56.4%)	47,551 (51.2%)
Missing	85 (0.1%)	5 (0.1%)	80 (0.1%)
	Most frequent principal psychiatric diagnoses		
	F33—22,535 (23.1%)	F33—1351 (29.7%)	F33—21,184 (22.8%)
	F20—13,915 (14.3%)	F31—1344 (29.6%)	F20—13,579 (14.6%)
	F32—11,073 (11.4%)	F25—697 (15.3%)	F32—10,876 (11.7%)
	F10—10,178 (10.5%)	F20—336 (7.4%)	F10—10,122 (10.9%)
	F05—4566 (4.7%)	F32—197 (4.3%)	F05—4551 (4.9%)
	F25—4556 (4.7%)	F60—197 (4.3%)	F25—3859 (4.2%)
	F31—4292 (4.4%)	F43—72 (1.6%)	F43—3720 (4.0%)
	F43—3792 (3.9%)	F10—56 (1.2%)	F31—2948 (3.2%)
	F60—3128 (3.2%)	F70—39 (0.9%)	F60—2931 (3.2%)
	F06—2084 (2.1%)	F71—34 (0.7%)	F06—2055 (2.2%)

Description of study population and patients with and without Li prescription by age groups, sex and most frequent *principal* psychiatric diagnoses. Psychiatric diagnoses by three character code of International Classification of Disease in its 10th Version, German Modification (ICD-10-GM): F33: Recurrent depressive disorder, F20: Schizophrenia, F32: Depressive episode, F10: Mental and behavioural disorders due to use of alcohol, F25: Schizoaffective disorders, F05: Delirium, not induced by alcohol and other psychoactive substances, F31: Bipolar affective disorder, F43: Reaction to severe stress, and adjustment disorders, F60: Specific personality disorders, F06: Other mental disorders due to brain damage and dysfunction and to physical disease, F70: Mild intellectual disability, F71: Moderate intellectual disability

### Prescription rate of lithium in selected psychiatric disorders

Patients with principal or secondary diagnosis of BD or UD constituted 69.7% of patients with Li prescription. 92.5% of patients with Li prescription either had a principal or secondary diagnosis of BD, SAD, UD or SCZ.

Out of the remaining 340 patients with Li prescription, 99 had a principal diagnosis of specific personality disorders (ICD-10-code F60) and 57 a principal diagnosis of mild (33 patients, ICD-10-code F70) or moderate (24 patients, ICD-10-code F71) intellectual disability. 33 patients had a principal diagnosis of reaction to severe stress, and adjustment disorders (ICD-10-code F43), 25 a principal diagnosis of other mental disorders due to brain damage and dysfunction and to physical disease (ICD-10-code F06) and 20 a principal diagnosis of pervasive developmental disorders (ICD-10-code F84).

Li prescription rate was highest in patients with principal diagnosis of BD (31.3%), followed by SAD (15.3%), UD (4.6%) and SCZ (2.4%).

**Fig. 1:**
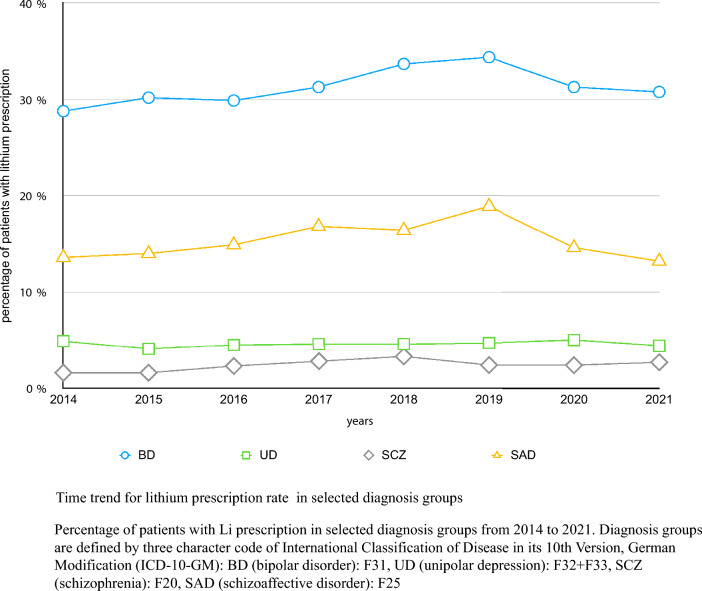
Time trend for lithium prescription rate in selected diagnosis groups

Prescription rates by year for BD, SAD, UD and SCZ are illustrated in Fig. 1.

In BD prescription rate increased significantly from 28.8% in 2014 to 34.4% in 2019 (χ^2^ = 3.96, p = 0.047) but gradually decreased to 30.8% thereafter. The change in prescription rate from 28.8% in 2014 to 30.8% in 2021 was not significant (χ^2^ = 0.49, p = 0.483). In UD, prescription rates remained stable over the observed period (χ^2^ = 3.08, p = 0.089).

In SCZ prescription rate increased significantly from 1.6% in 2014 to 3.3% in 2018 (χ^2^ = 10.66, p = 0.001). The following years showed lower prescription numbers. The increase in prescription rate from 1.6% in 2014 to 2.7% in 2021 was significant (χ^2^ = 5.37, p = 0.020).

In SAD prescription rate increased significantly from 13.6% in 2014 to 18.9% in 2019 (χ2 = 5.94, p = 0.015). After 2019 numbers gradually decreased to 13.2% in 2021. The decrease in prescription rate from 13.6% in 2014 to 13.2% in 2021 was not significant (χ2 = 0.05, p = 0.832).

### Lithium prescription in young women, older patients and patients with comorbid substance use disorder

In the study population, prevalence of comorbid substance use disorder was 13.4% in BD, 15.1% in UD, 17.0% in SCZ and 11.9% in SAD. In patients with Li prescription prevalence of comorbid substance use disorder was 12.4% in BD, 11.9% in UD, 17.0% in SCZ and 10.0% in SAD.

Table 3 presents Li prescription numbers along with calculated relative risks in women under 41 years compared to men under 41 years, in adults older than 65 years compared to those 65 years and younger and in patients with comorbid substance abuse disorder compared to patients without comorbid substance abuse disorder.

**Table 3:** Lithium prescription in young women, older adults and comorbid substance use disorder

	Bipolar disorder	Unipolar depression	Schizophrenia	Schizoaffective disorder
Women under 41 years	174/495 35.2%	230/6298 3.7%	48/1834 2.6%	119/728 16.3%
Men under 41 years	144/407 35.4%	178/4690 3.8%	87/4368 2.0%	74/488 15.2%
RR with 95% CI	0.99 (0.83 to 1.19)	0.96 (0.79 to 1.17)	1.31 (0.93 to 1.86)	1.31 (0.93 to 1.86)
Older than 65 years	221/941 23.5%	290/6208 4.7%	11/1257 0.9%	76/621 12.2%
65 and younger	1121/3344 33.5%	1257/27356 4.6%	325/12652 2.5%	621/3933 15.8%
RR with 95% CI	0.70 (0.62 to 0.79)	1.02 (0.90 to 1.15)	0.34 (0.19 to 0.62)	0.78 (0.62 to 0.97)
Comorbid substance use disorder	166/576 28.8%	184/5077 3.6%	57/2360 2.4%	70/542 12.9%
No comorbid substance use disorder	1178/3716 31.7%	1364/28531 4.8%	279/11555 2.4%	627/4014 15.6%
RR with 95% CI	0.91 (0.79 to 1.04)	0.76 (0.65 to 0.88)	1.00 (0.75 to 1.33)	0.83 (0.66 to 1.04)

Absolute and relative prescription numbers of Li, along with calculated Relative Risks and 95% CI in women under 41 vs. men under 41, patients older than 65 years vs. patients 65 and younger and patients with comorbid substance abuse disorder vs. patients without comorbid substance abuse disorder. Diagnosis groups are defined by three-character code of International Classification of Disease in its 10th Version, German Modification (ICD-10-GM): Bipolar disorder: F31, Unipolar depression: F32 + F33, Schizophrenia: F20, Schizoaffective disorder: F25 Comorbid substance abuse disorder was defined by ICD-10-diagnosis of mental and behavioral disorders due to psychoactive substance use, excluding tobacco use (F10-F19, excluding F17)

### Comorbidities that constitute relative or absolute contraindication for Lithium prescription

Comorbidities that constitute relative or absolute contraindication for Li prescription, as defined by the section “Recommendation for Li use” in the German 2019 S3 guidelines for BD ( DGBS e.V. und DGPPN e.V. 2019), were present in 2.2% (n = 2165) of the study population. In patients with Li prescription, those comorbidities were present in 1.5% (n = 69) of patients. Two of 182 pregnant patients were prescribed Li.

In patients with Li prescription, six absolute contraindications were recorded. Two cases of acute myocardial infarction and four cases of acute renal failure. Additionally, eight cases of unspecified kidney failure were recorded.

The most prevalent relative contraindication was chronic kidney disease with 33 cases. 22 cases of psoriasis were recorded.

### Comedication with risk of drug–drug interactions

On average, patients in the study population were prescribed a number of 4.81 drugs (SD = 3.49), patients with Li 6.14 drugs (SD = 3.13), significantly more than Non-Li patients (p < 0.001). 45% of the study population were prescribed 5 drugs or more. In patients with Li prescription 64% were prescribed 5 drugs or more.

178 prescriptions with mediQ high-priority drug–drug interaction with Li were identified (mediQ-Interaktionsdatenbank 2023). The diuretic hydrochlorothiazide (n = 157) was the most administered drug with high-priority drug–drug interaction, followed by the angiotensin II receptor antagonist olmesartan (n = 16), and the thiazide-like diuretics indapamide (n = 4) and chlortalidone (n = 1). For all prescribed diuretics with high-priority drug–drug interaction, mediQ states increased risk for Li intoxication caused by increased reuptake of Li and sodium in the proximal tubular cells and consequently elevated Li blood levels. For olmesartan it states risk for reversible increase in Li blood levels and therefore possible Li toxicity.

Hydrochlorothiazide was the 37th most prescribed comedication for Li. Within the 50 most prescribed comedications, there were also 20 drugs with intermediate-priority drug–drug interactions with Li and 17 drugs with low-priority drug–drug interactions with Li, as classified by the drug–drug interaction program mediQ.

### Severe adverse drug reactions with lithium as the reported probable causative agent

From 2016 to 2021 in 1.2% of all patients (859 out of 73,533) one or more sADRs were reported. In patients with Li prescription 2.0% of patients (70 out of 3416) were reported to have experienced sADRs.

30 sADRs in 23 patients were reported with Li as the reported probable causative agent. We excluded 4 patients, where no measures were taken as a consequence of the adverse reaction. 19 patients with 26 reported sADRs remained. 11 patients were female, eight male. Four patients were older than 65 years. Six affected patients had a diagnosis of UD, eight of BD and five of SAD. Six patients were diagnosed with comorbid substance use disorder (three cases of alcohol dependence, three cases of sedative, hypnotic or anxiolytic-related dependence). Mean number of drugs prescribed was 6.47 (SD 2.61).

Table 4 presents all 19 patients with reported sADRs, including individual sADR, Li serum levels if provided, comedication with possible drug–drug interactions and measures taken as consequence of sADR. SADRs are ordered by severity of measures taken.

**Table 4:** Severe adverse drug reactions with lithium as reported probable causative agent 2016 to 2021

sADR and lithium serum level if provided	Comedication with high or intermediate drug–drug interaction	Measures taken
1. Li-intoxication (agitation/confusion) 2. Tremor 3. Nausea, vomiting	h: Hydrochlorothiazide i: Ramipril	• Transfer to ICU • Discontinuation of Li and Hydrochlorothiazid
1. Blurred vision 2. Diarrhea 3. Vertigo Li serum level: 2.21 mmol/	i: Haloperidol, Clozapine, Enalapril	• Transfer to internal medicine • Discontinuation of Li
Agitation Li serum level: 1.36 mmol/l	i: Olanzapine, Valsartan	• Hospitalization • Discontinuation of Li
1. Psychomotor retardation 2. EPS	i: Doxepine, Haloperidol	• Biperiden application • Discontinuation of Li
1. Cognitive deficits 2. diarrhea Li serum level: 1.56 mmol/l	i: Diltiazem, Candesartan	• Discontinuation of Li • Reduction of Candesartan
Li-intoxication (restlessness/agitation)	i: Ramipril, Flupentixol	• Discontinuation of Li
Renal dysfunction	i: Clozapine, Haloperidol	• Discontinuation of Li
Renal dysfunction	h: Hydrochlorothiazide i: Duloxetine, Valsartan, Pipamperone	• Discontinuation of Li
Tremor	i: Sertraline	• Discontinuation of Li
1. Tremor 2. Nausea, vomiting	i: Venlafaxin, Flupentixol	• Reduction of Li and Mirtazapine • discontinuation of Flupentixol
nausea and vomiting	i: Venlafaxine, Pipamperone	• Reduction of Li dosage
Tremor	i: Piroxicam	• Reduction of Li dosage
Tremor	i: Ramipril	• Reduction of Li dosage
Tremor	i: Ramipril, Duloxetin	• Reduction of Li dosage
Tremor	i: Venlafaxine	• Reduction of Li dosage
Tremor	i: Tapentadol, Etoricoxib, Macrogol	• Reduction of Li dosage
Edema of hands, feet, face	i: Venlafaxine	• Reduction of Li dosage
Renal dysfunction	i: Venlafaxine, Amisulpride	• Consultation with internal medicine
QTc-elongation	i: Duloxetine, Pipamperone	• Ecg control

Reported severe adverse drug reactions along with comedication with possible drug–drug interactions and measures taken as consequence of sADR. sADRs are ordered by severity of measures taken. h: drugs with high-priority drug–drug interactions, i: drugs with intermediate-priority drug–drug interactions. Drugs were classified using mediQ-database. MediQ-search (https://www.mediq.ch) was last reviewed May 08, 2023

## Discussion

### Lithium prescription in mood disorders and other diagnosis groups

The present study demonstrates that utilization of Li in clinical practice far exceeds acute and prophylactic treatment in BD and UD. Almost one in three patients with Li prescription did not have diagnosis of BD or UD. More than one in five patients with Li prescription had a diagnosis of SAD or SCZ, even though evidence for effectiveness of Li use in monotherapy or as an adjunct to antipsychotics in SCZ is scarce (Leucht et al. 2015) and 2019 German S3 guidelines for SCZ, which no longer recommend Li for treatment of depressive symptoms in SCZ, even advise against its use as an augmentative in standard treatment for improvement of general or affective symptoms and aggression (DGPPN e.V. (Hrsg.) 2019). Clinicians might use Li in a symptom-oriented approach in patients with mood disorders that do not meet criteria for diagnosis of BD or UD. Another rationale, especially for administration of Li in patients with specific personality disorders or intellectual disability, could be the positive effects of Li on impulsive and violent behavior (Müller-Oerlinghausen and Lewitzka 2010).

### Lithium in bipolar disorder

Internationally, prescription rates and trends in prescription differ widely (Singh et al. 2023). For example, in Sweden, Li prescription rates in outpatients with BD decreased from 51% in 2007 to 41% in 2013 (Karanti et al. 2016), while in the United States, data from the National Ambulatory Medical Care Surveys shows that Li prescription rates in BD decreased from 30.4% in 1997 to 17.6% in 2016 (Rhee et al. 2020).

Our study’s overall Li prescription rate of 31.3% in patients with BD seems to support previous findings about a steady decline in Li utilization in BD in Germany, as an analysis of data from the German Drug Safety Program in Psychiatry (AMSP) showed a decrease in Li prescription rates in selected German hospitals from 44.8% in 1994 to 34.4% in 2009 (Greil et al. 2012).

The AMSP Drug Safety project is designed similar to the Pharmako-EpiVig project and also collects data about drug use and sADRs on two reference days a year from more than 30 hospitals in Germany, Switzerland and Austria (Grohmann et al. 2004).

However, prescription rate in BD over the course of our study significantly increased from 28.8% in 2014 to 34.4% in 2019. Only after 2019 the data shows a decline in prescription rate to 30.8% in 2021.

This decline in prescription numbers after 2019 must be considered in light of the COVID-19 pandemic, as the pandemic and consequent policy responses influenced access to health care services. Because of the pandemic, in early 2020 there was a limited availability of inpatient treatment capacity and outpatient services in psychiatric hospitals in Germany (Adorjan et al. 2021). In Li treatment, unhindered access to health care services is a prerequisite, due to the need for regular blood level monitoring. Practitioners as well as patients might therefore have been reluctant to initiate or continue Li treatment because of restrictions associated with the COVID-19 pandemic.

Despite availability of alternative treatment options, like second generation antipsychotics and other anticonvulsants, Li should be strongly considered for every patient with BD in absence of contraindications. Li is still the only drug with a level A recommendation as a mood stabilizer in long-term treatment of BD by the 2019 German S3 guidelines for BD and evidence regarding its efficacy in treatment of bipolar disorder has been strengthening over the last decade ( DGBS e.V. und DGPPN e.V. 2019; Severus et al. 2014; Bschor et al. 2020). Additionally, its unique anti-suicidal effects can mitigate the risk for suicide in patients with major affective disorders (Cipriani et al. 2013; Lewitzka et al. 2015; Tondo et al. 2001).

Since this study includes patients at all time points of course of disease, a considerable number of patients that were not prescribed Li on the reference day, might have been treated with Li in the past and have discontinued treatment due to side effects, personal preferences or poor response. Depending on clinical, biological and genetic features, inter-individual response in Li varies substantially, full response is observed in about 30% of patients with BD (Hou et al. 2016; Tighe et al. 2011; Bauer and Gitlin 2016b). Therefore, comparisons to findings about prescription rate of Li in other studies must be done cautiously.

Lastly, changes in prescription rates over the observed period might also be influenced by differences in the study population at given reference days, i.e. proportion of patients with manic vs. depressive symptoms or type I vs. type II subtypes.

### Lithium in UD

Overall, Li prescription rate in UD was 4.6% and remained stable over the observed time period. AMSP analysis showed similar prescription rates of about 4.9% in the years 2015 to 2017 (Seifert et al. 2021).

Augmentation treatment, as an add-on to an antidepressant in treatment-resistant depression is Li’s main indication in UD. German 2022 S3 guidelines on UD and several international guidelines, recommend Li as first-line therapy in treatment-resistant depression (Bundesärztekammer (BÄK) et al. 2022; Taylor et al. 2020). However, recent studies and metanalyses confirm profound efficacy and safety of several second-generation antipsychotics as an alternative in adjunctive therapy, on a similar or even superior evidence base when compared to Li, contributing to a decline in Li prescription (Marcus et al. 2008; Nuñez et al. 2022).

In contrast to numerous publications about Li prescription practice in BD, studies about prescription practice of Li in UD are hardly available. This restricts interpretation of our findings and demonstrates further need of research in this area.

### Lithium in older patients

Patients older than 65 years had significantly lower probability to be treated with Li in BD, SCZ and SAD. Lower utilization of Li in older patients in BD has been reported before (Rej et al. 2017).

In old age Li is still considered first choice for maintenance-treatment in BD (Volkmann et al. 2020). Li not only mitigates the manifold increased risk of suicide in patients with BD, but also reduces excess cardiovascular mortality (Lewitzka et al. 2015; Ahrens et al. 1995). Long-term treatment reduces frequency of psychiatric as well as somatic hospitalization (Lähteenvuo et al. 2023).

Patients with BD suffer from extensive medical comorbidity, standardized mortality is about twice as high as in the general population (Westman et al. 2013; Walker et al. 2015). Therefore, treatment options in older patients can be limited by somatic comorbidity (e.g. kidney failure) due to reduced drug tolerability and altered drug-metabolism. Also there might be a reluctance to prescribe Li in elderly patients due to increased vulnerability to Li intoxication, even though this risk can be mitigated by lowering dosage and intensifying serum level controls (Gitlin 2016b). Lastly, since onset of BD, SCZ and SAD is typically in young age, older patients might simply have been treated with Li in the past and discontinued it due to poor response or side effects. Studies suggest that in long-term treatment up to more than half of patients with BD ore SAD decide to discontinue Li at some point (Öhlund et al. 2018).

It is notable, that in UD, old age did not have an influence on prescription rate.

### Patients with comorbid substance use disorders

Concomitant substance use disorders are common in severe psychiatric disorders (Singh et al. 2023; Davis et al. 2008). To our best knowledge, there are no previous studies about Li prescription rate in patients with comorbid substance use disorders. Knowledge about Li therapy in patients with comorbid substance use is limited, since substance use disorders are common exclusion criteria in randomized trials. While some studies associated alcohol use disorders with poor response to Li (Sportiche et al. 2017; Grillault Laroche et al. 2020), a recent systematic review concludes that valproate and lamotrigine should preferably be used in BD with concomitant substance abuse disorder but emphasizes poor quality of evidence and need for further research (Coles et al. 2019).

Even though substance use disorders are linked to poor adherence in patients with severe mental illness (García et al. 2016), and therefore this subgroup of patients might be more exposed to the specific risks of poor adherence such as Li toxicity and rebound suicidality after abrupt Li cessation, prescription rate of Li in patients with comorbid substance use disorder in our study was only significantly lower in UD.

### Comorbidities that constitute relative or absolute contraindication for lithium prescription

Prevalence of somatic comorbidities that constitute relative or absolute contraindications for Li prescription was 1.5% in patients with Li prescription and 2.2% in the study population. Due to differences in psychiatric morbidity and consequent sociodemographic heterogeneity, comparison between those groups is only reasonable to a very limited extend. Reported absolute contraindications (two cases of acute myocardial infarction and four cases of acute renal failure), were not reported as sADRs, so it is probable that time of diagnosis preceded the day of the survey by at least 2 weeks. Most prevalent contraindications were acute or chronic renal failure and psoriasis.

Renal failure and psoriasis can be caused and aggravated by Li treatment, but neither is a determinant reason to discontinue Li treatment. An option for managing psoriasis can be lowering dosage of Li. In most cases Li-associated renal effects are relatively mild and progressive renal impairment due to long-term Li use can be monitored by regular blood tests. Psychiatric disorders are no less debilitating in terms of quality of life and mortality compared to numerous other chronic medical conditions. A prime example is rheumatic diseases. In this case as well, within the framework of guideline-compliant treatment, the risks and benefits (e.g., with chemotherapy) need to be carefully weighed against the potential side effects. Li treatment also requires a constant monitoring of response and side effects. In absence of response Li should be discontinued. In patients that benefit from Li therapy, it is not a trivial task to weigh the relief of highly debilitating, sometimes life-threatening affective symptoms against the risks of aggravation of already perceivable long-term side effects, that might lead to severe disability like need for lifelong renal replacement therapy (Gitlin 2016b; Jafferany 2008; Tondo et al. 2017).

### Comedication with risk of drug–drug interactions

Patients with Li prescription were prescribed significantly more drugs than patients without Li prescription. As mentioned above, due to differences in psychiatric morbidity and consequent sociodemographic heterogeneity comparison between those groups is limited. Almost two out of three patients with Li prescription were prescribed five drugs or more simultaneously.

For classification of drug–drug interactions with Li, we used the mediQ database which in a recent study has been evaluated as the most suitable interaction database for psychopharmacotherapy (Hahn and Roll 2018).

Prescribed drugs with highly relevant drug–drug interactions (n = 178) were the diuretics hydrochlorothiazide (n = 157), indapamide (n = 4) and chlortalidone (n = 1), and the antihypertensive olmesartan (n = 16). Since these diuretics as well as olmesartan increase Li blood levels by increased reuptake in the kidneys, acute and chronic toxicity of Li can be increased. However, with reduction of dosage and regular monitoring of serum concentration, this interaction can be well managed (Malhi et al. 2020).

### Severe adverse drug reactions with lithium as the reported probable causative agent

Even though underreporting cannot be ruled out, considering the total number of 4543 patients treated with Li, a figure of 19 patients with sADRs seems comparatively low. This suggests a high level of safety for Li therapy in inpatient settings.

Almost one in three patients with reported sADRs had diagnosis of comorbid substance use disorder. This suggests that patients with comorbid substance use disorder are more vulnerable to adverse drug reactions. Almost three in four patients were prescribed at least two comedications with intermediate- or high-priority drug–drug interaction with Li. Four patients were reported to have lithium intoxications with levels ranging from 1.3 up to 2.2 mmol/l. Of these, one patient, who had received hydrochlorothiazide as comedication, had to be transferred to the intensive care unit. The other patients received blood pressure drugs such as ACE inhibitors or sartans. One patient in which Li had to be discontinued due to symptoms of Li intoxication (restlessness/agitation) was prescribed Flupentixol. Some studies suggest that phenothiazines might increase intracellular Li concentration (Pandey et al. 1979).

Tremor is the most common reason for Li discontinuation, therefore it is important to discuss this and other common side effects, like weight gain before the start of treatment, so counter measures can be taken, and unnecessary discontinuation prevented (Öhlund et al. 2018; McCreadie et al. 1985).

On the other hand, it should also be mentioned that many alternatives to Li in treatment of affective disorders have their own inherent side effect profile. Alternatives for maintenance-treatment like valproate, olanzapine or quetiapine, for example, have much more unfavorable metabolic side effects than Li (Greil et al. 2023).

While it is known that multiple drugs have additive effect on side effect rate in Li therapy (Gitlin 2016b), it is still remarkable that in all cases of the most severe sADRs, comedications with drugs that increase Li serum levels, namely hydrochlorothiazide, ramipril, enalapril, valsartan and candesartan were prescribed. These findings emphasize the significance of attention to drug–drug interaction in psychopharmacology in general and Li therapy in particular.

### Strengths and limitation

Data for this study was collected in a repeated cross-sectional approach, therefore the study design does not allow to draw conclusions about causal relationship of findings. No information was available about patient’s treatment history or course of diseases restricting interpretation of findings. Due to the use of ICD-10 coding, a distinction between bipolar 1 and bipolar 2 disorders was not made. Consequently, separate analyses for both conditions regarding prescription frequency were not conducted. Furthermore, the diagnosis F30 was excluded from definition of BD. Since Li is also approved for the treatment of mania in Germany, it is possible that this may have led to an overall underestimation of prescription rates. Because of to the design of this deadline survey, which only captures severe adverse events appearing within the two weeks before the deadline the incidence of severe adverse events may be underestimated. Since the source of this study is an observational database, there is possibility of underreporting, missing or incorrect information about diagnoses, drug prescriptions or sADRs. Due to the naturalistic nature of the data and the exploratory approach of the analysis, we refrained from using more elaborate statistical methods, which also includes correction for multiple testing.

However, the quasi-naturalistic setting and inclusion of all patients present at the included hospitals, made it possible to demonstrate the full picture of current Li prescription practice in inpatient care in Bavaria.

## Conclusion

Our study does not substantiate concerns about further decline in Li prescription in BD and UD. While prescription numbers were stable in UD, we saw a significant increase in Li prescription in BD from 2014 to 2019. Decreasing numbers in 2020 and 2021 might be associated with the COVID-19 pandemic.

The fact that almost a third of patients with Li prescription were not diagnosed with BD or UD suggests that clinicians also experience positive results of symptom-oriented utilization of Li in other diagnosis groups. This emphasizes the need for further scientific exploration of Li's efficacy in treatment of patients with symptoms of mood disorders that do not meet criteria for UD or BD.

Prevalence of patients older than 65 and patients with comorbid substance use disorder in patients with Li prescription was considerable and demonstrates the need for further studies about safety and efficacy of Li in these groups. Equal prescription rates in patients younger and older than 65 years in UD suggest that there is no general reluctance of clinicians to prescribe Li in elderly patients. Similarly, comorbid substance use disorder does not seem to be a major determent to initiation of Li therapy, with only significantly lower prescription rate in this group in UD.

Polypharmacy was present in almost two thirds of patients with Li prescription. Our study demonstrates that possible drug–drug interactions with Li are described for the majority of commonly prescribed comedications. Reported cases of sADRs underline the relevance of drugs that can influence Li serum levels.

## List of repeatedly referred to diagnoses with corresponding defined code of the ICD-10 classification system

**Table:** 

Comorbid substance use disorder	F10-F19, excluding F17
Schizophrenia	F20
Schizoaffective disorder	F25
Bipolar disorder	F31
Unipolar depression	F32 or F33
Specific personality disorders	F60
Mild intellectual disability	F70
Moderate intellectual disability	F71
Primary adrenocortical insufficiency	E27.21
Addisonian crisis	E27.2
Acute myocardial infarction	I21
Subsequent myocardial infarction	I22
Psoriasis	L40
Acute renal failure	N17
Chronic kidney disease	N18
Unspecified kidney failure	N19

### Supplementary Information

**Additional file 1: Table S1.** List of participating hospitals 2014 to 2021. **Table S2.** Prescription numbers of lithium by year and diagnosis group. **Table S3.** Top 20 drugs with intermediate-priority drug–drug interactions with lithium. Pharmako-EpiVig survey questionnaire.

## Data Availability

The data that support the findings of this study are available from Bavarian Institute for Data, Analysis and Quality Assurance (BIDAQ), Am Moosfeld 13, 81829 München, E-Mail: kontakt@bidaq.de, but restrictions apply to the availability of these data, which were used under license for the current study, and so are not publicly available. Data are however available from the authors upon reasonable request and with permission of Bavarian Institute for Data, Analysis and Quality Assurance (BIDAQ).

## Abbreviations

BD: Bipolar disorder
BIDAQ: Bavarian Institute for Data, Analysis and Quality Assurance
Li: Lithium
SAD: Schizoaffective disorder
sADR: Severe adverse drug reaction
SCZ: Schizophrenia
SD: Standard deviation
UD: Unipolar depression
