# Supplementary material for: Lithium prescription trends in psychiatric inpatient care 2014 to 2021: data from a Bavarian drug surveillance project
Source: Int J Bipolar Disord. 2023 Dec 19;11:40. doi: 10.1186/s40345-023-00323-6 (PMC10730486; doi:10.1186/s40345-023-00323-6)
Supplement: Supplementary file 1 — Additional file 1: Table S1. List of participating hospitals 2014 to 2021. Table S2. Prescription numbers of lithium by year and diagnosis group. Table S3. Top 20 drugs with intermediate-priority drug–drug interactions with lithium. Pharmako-EpiVig survey questionnaire. [file 40345_2023_323_MOESM1_ESM.docx]

**Additional file**

| **Table S1 List of participating hospitals 2014 to 2021** | | | | | | | | |  |
| --- | --- | --- | --- | --- | --- | --- | --- | --- | --- |
|  | *years* | | | | | | | |  |
|  | 2014 | 2015 | 2016 | 2017 | 2018 | 2019 | 2020 | 2021 | |
|  |  |  |  |  |  |  |  |  | |
| Bezirksklinik Hochstadt | x | x | x | x | x | x | x | x | |
| Bezirksklinikum Ansbach | x | x | x | x | x | x | x | x | |
| Bezirksklinikum Mainkofen | x | x | x | x | x | x | x | x | |
| Bezirksklinikum Obermain | x | x | x | x | x | x | x | x | |
| Bezirksklinikum Regensburg |  |  | x | x | x | x | x | x | |
| Bezirksklinik Rehau | x | x | x | x | x | x | x | x | |
| Bezirkskrankenhaus Augsburg | x | x | x | x | x | x | x | x | |
| Bezirkskrankenhaus Bayreuth | x | x | x | x | x | x | x | x | |
| Bezirkskrankenhaus Günzburg | x | x | x | x | x | x | x | x | |
| Bezirkskrankenhaus Kaufbeuren | x | x | x | x | x | x | x | x | |
| Bezirkskrankenhaus Kempten | x | x | x | x | x | x | x | x | |
| Bezirkskrankenhaus Landshut | x | x | x | x | x | x | x | x | |
| Bezirkskrankenhaus Lohr | x | x |  |  |  |  |  |  | |
| Bezirkskrankenhaus Memmingen | x | x | x | x | x | x | x | x | |
| Frankenalb-Klinik Engelthal | x | x | x | x | x | x | x | x | |
| kbo-Isar-Amper-Klinikum München Ost | x | x | x | x | x | x | x | x | |
| kbo-Inn-Salzach-Klinikum | x | x | x | x | x | x | x | x | |
| kbo-Isar-Amper-Klinikum Taufkirchen (Vils) | x | x | x | x | x | x | x | x | |
| kbo-Lech-Mangfall-Klinik Agatharied | x | x | x | x | x | x | x | x | |
| kbo-Lech-Mangfall-Klinik Garmisch-Partenkirchen | x | x | x | x | x | x | x | x | |
| kbo-Lech-Mangfall-Klinik Landsberg am Lech | x | x | x | x | x | x | x | x | |
| Klinikum am Europakanal | x | x | x | x | x | x | x | x | |
| Krankenhaus für Psychiatrie, Psychotherapie und Psychosomatische Medizin Schloss Werneck | x | x | x | x | x | x | x | x | |
| Tagesklinik Lindau | x | x | x | x | x | x | x | x | |
| Bezirkskrankenhaus Donauwörth |  |  | x | x | x |  |  |  | |
| Hospitals participating in the Pharmako-EpiVig survey by year 2014 to 2021 | | | | | | | | |  |

| **Table S2 Prescription numbers of lithium by year and diagnosis group** | | | | |
| --- | --- | --- | --- | --- |
| *Year* | *bipolar disorder* | *unipolar depression* | *schizophrenia* | *schizoaffective disorder* |
| *2014* | 155/539  28.8% | 206/4166  4.9% | 27/1706  1.6% | 81/595  13.6% |
| *2015* | 175/579  30.2% | 179/4317  4.1% | 28/1722  1.6% | 84/601  14.0% |
| *2016* | 158/529  29.9% | 200/4412  4.5% | 41/1774  2.3% | 89/598  14.9% |
| *2017* | 181/579  31.3% | 202/4347  4.6% | 51/1792  2.8% | 91/541  16.8% |
| *2018* | 197/585  33.7% | 209/4561  4.6% | 58/1758  3.3% | 97/591  16.4% |
| *2019* | 188/547  34.4% | 209/4447  4.7% | 43/1768  2.4% | 109/578  18.9% |
| *2020* | 142/453  31.3% | 175/3518  5.0% | 42/1716  2.4% | 78/536  14.6% |
| *2021* | 148/481  30.8% | 168/3840  4.4% | 46/1679  2.7% | 68/516  13.2% |
| *Total* | 1344/4292  31.3% | 1548/33608  4.6% | 336/13915  2.4% | 697/4556  15.3% |
| Absolute and relative prescription numbers of LI in selected *principal* diagnosis groups from 2014 to 2021. Diagnosis groups are defined by three-character code of International Classification of Disease in its 10th Version, German Modification (ICD-10-GM): bipolar disorder: F31, unipolar depression: F32+F33, s*chizophrenia: F20*, schizoaffective disorder: F25. | | | | |

| Table S3 Top 20 drugs with intermediate-priority drug-drug interactions with lithium | | |
| --- | --- | --- |
| *drug* | *n* | *mediQ comments about interaction* |
| Venlafaxine | 799 | increased risk for serotonin toxicity |
| Olanzapine | 784 | increased risk for neurotoxicity, lowering of seizure threshold, EPS, QT-elongation, potentiation of ADRs like weight gain, hyperglycemia, sleepiness, tremor, slurred speech |
| Risperidone | 569 | increased risk for neurotoxicity, lowering of seizure threshold, EPS, QT-elongation |
| Pipamperone | 487 | increased risk for lowering of seizure threshold, EPS, QT-elongation and neurotoxicity rare cases of enzephalopathic syndromes |
| Sertraline | 478 | increased risk for serotonin toxicity, QT-elongation, influence on sodium balance, bodyweight and glucose-regulation |
| Ramipril | 475 | increase of lithium blood levels |
| Duloxetin | 283 | increased risk for serotonin toxicity, potentiation of ADRs like hyponatremia and hypothyroidism |
| Clozapine | 278 | increased risk for neurotoxicity, weight gain, cardiovascular ADRs i.e., QT-elongation, potential of lithium to increase neutrophil cell count in benign neutropenia |
| Haloperidol | 263 | increased risk for QT-elongation, neurotoxicity, EPS, rare cases of enzephalopathic syndromes |
| Prothipendyl | 232 | increased risk for QT-elongation, neurotoxicity, EPS, rare cases of enzephalopathic syndromes |
| Amitriptyline | 195 | increased risk for QT-elongation, neurotoxicity, lowering of seizure threshold |
| Chlorprothixene | 161 | increased risk for QT-elongation, EPS, rare cases of enzephalopathic syndromes |
| Zuclopenthixol | 160 | increased risk for lowering of seizure threshold, QT-elongation and neurotoxicity |
| Amisulpride | 152 | increase of Amisulpride blood levels, increased risk for QT-elongation, neurotoxicity, EPS |
| Escitalopram | 147 | increased risk for serotonin toxicity, QT-elongation |
| Promethazine | 142 | increased risk for QT-elongation, neurotoxicity, EPS, enzephalopathic syndromes, lowering of seizure threshold |
| Candesartan | 138 | increase of lithium blood levels |
| Flupentixol | 136 | increased risk for QT-elongation, neurotoxicity, EPS, rare cases of enzephalopathic syndromes |
| Torasemide | 128 | increase of lithium blood levels |
| Metamizole | 125 | possible increase of lithium blood levels |
| 20 most commonly prescribed drugs in patients with LI prescription with intermediate-priority drug-drug interactions with LI, along with, prescription numbers and mediQ comments about interaction risk. MediQ-search (https://www.mediq.ch) was last reviewed January 19, 2022 | | |

**Pharmako-EpiVig survey questionnaire**


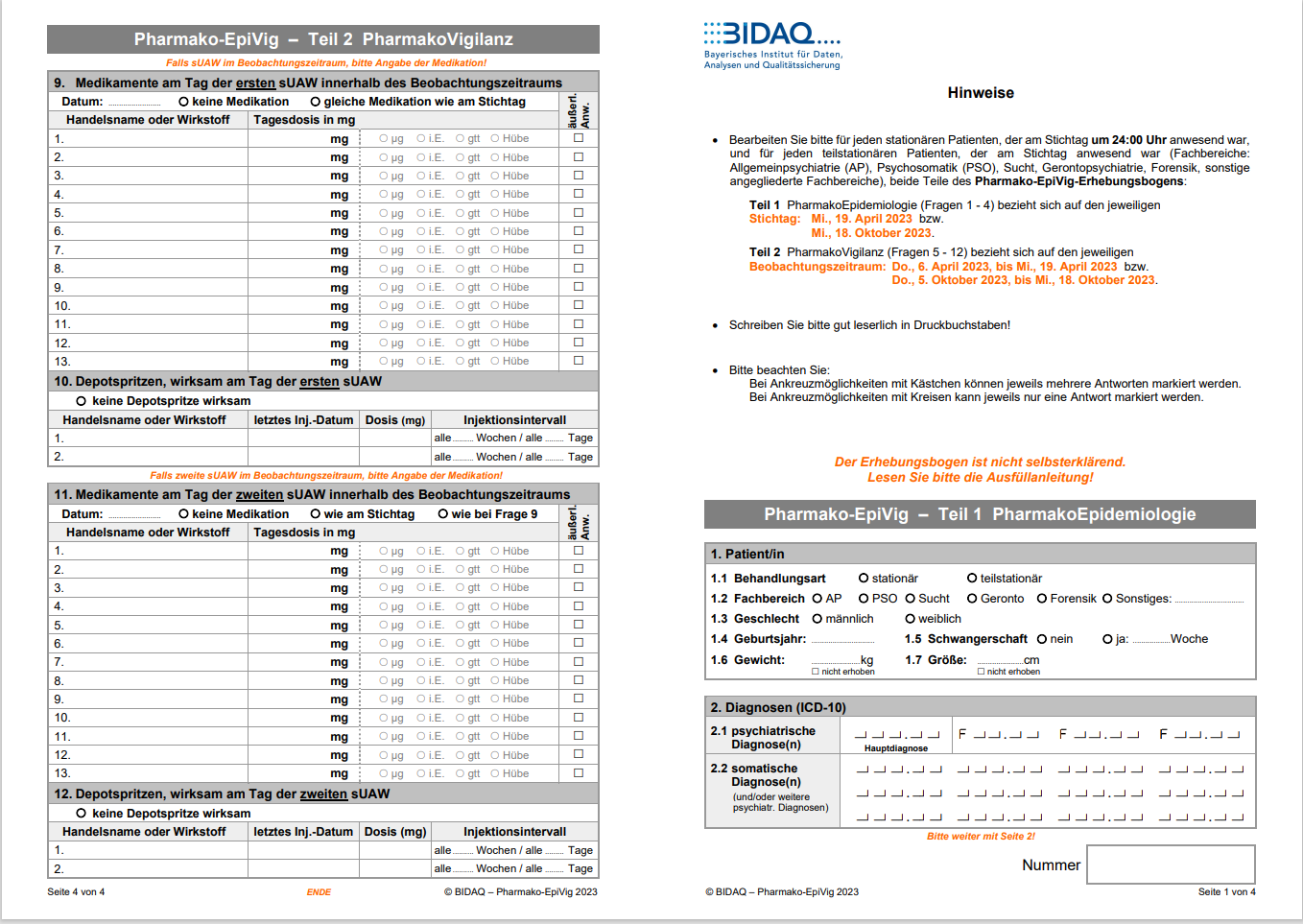


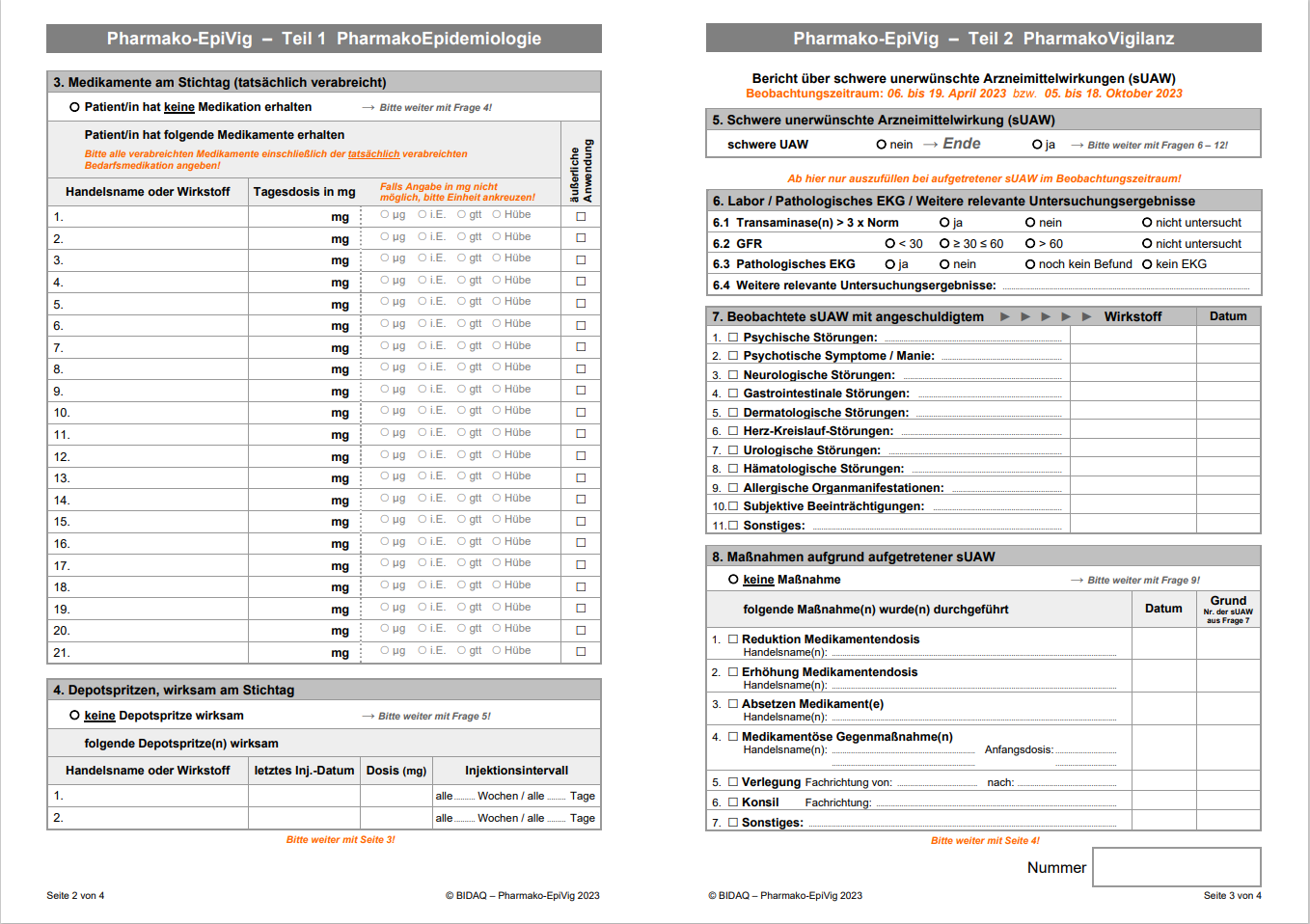


Questionnaire and associated instructions can be found on the BIDAQ-homepage under <https://bidaq.de/projekte/epivig/dokumente>.
